# Genome sequence of the halotolerant bacterium *Corynebacterium halotolerans* type strain YIM 70093^T^ (= DSM 44683^T^)

**DOI:** 10.4056/sigs.3236691

**Published:** 2012-12-18

**Authors:** Christian Rückert, Andreas Albersmeier, Arwa Al-Dilaimi, Karsten Niehaus, Rafael Szczepanowski, Jörn Kalinowski

**Affiliations:** 1 Technology Platform Genomics, CeBiTec, Bielefeld University, Bielefeld, Germany; 2 Proteome and Metabolome Research, Bielefeld University, Bielefeld, Germany

**Keywords:** aerobic, non-motile, Gram-positive, mesophilic, halotolerant

## Abstract

*Corynebacterium halotolerans* Chen *et al*. 2004 is a member of the genus *Corynebacterium* which contains Gram-positive bacteria with a high G+C content. *C. halotolerans*, isolated from a saline soil, belongs to the non-lipophilic, non-pathogenic corynebacteria. It displays a high tolerance to salts (up to 25%) and is related to the pathogenic corynebacteria *C. freneyi* and *C. xerosis*. As this is a type strain in a subgroup of *Corynebacterium* without complete genome sequences, this project describing the 3.14 Mbp long chromosome and the 86.2 kbp plasmid pCha1 with their 2,865 protein-coding and 65 RNA genes will aid the *** G****enomic* ***E****ncyclopedia of* ***Bacteria**** and* ***Archaea***** project.

## Introduction

Strain YIM 70093^T^ (= DSM 44683^T^) is the type strain of the species *Corynebacterium halotolerans* [1] and was originally isolated from saline soil in Xinjiang Province in western China. The genus *Corynebacterium* is comprised of Gram-positive bacteria with a high G+C content. It currently contains over 80 members [2] isolated from diverse backgrounds like human clinical samples [3] and animals [4], but also from soil [5] and ripening cheese [6].

Within this diverse genus, *C. halotolerans* has been proposed to form a subclade together with *C. freneyi* and *C. xerosis* [1]. Data concerning salt tolerance is not available for most corynebacteria, but *C. halotolerans* YIM 70093^T^ displays the highest resistance to salt (up to 25%) described for *Corynebacterium* so far. Here we present a summary classification and a set of features for *C. halotolerans* YIM 70093^T^, together with the description of the genomic sequencing and annotation.

## Classification and features

A representative genomic 16S rRNA sequence of *C. halotolerans* YIM 70093^T^ was compared to the Ribosomal Database Project database [7], confirming the initial taxonomic classification. Addition of the recently published species *C. maris* Coryn-1^T^ [8], *C. marinum* 7015^T^ [9] and *C. humireducens* MFC-5^T^ [10] as well as *C. diphtheriae* NCTC 11397^T^ [11] indicates that *C. halotolerans* YIM 70093^T^, together with *C. maris*, *C. marinum*, and *C. humireducens*, form a distinct subclade within the genus *Corynebacterium*. Interestingly, *C. xerosis* and *C. freneyi* do not group closely with this subclade when *C. diphtheriae* is added to the comparison.

**Figure 1:**
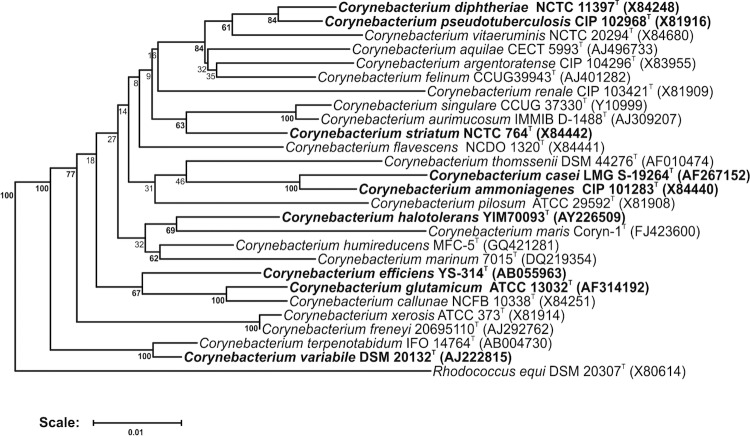
Phylogenetic tree highlighting the position of *C. halotolerans* relative to type strains of other species within the genus *Corynebacterium* as selected by Chen *et al.* [1]. In addition, the recently described *C. maris*, *C. marinum*, and *C. humireducens* were added, as they were shown to be closely related. Furthermore, the type strain of the genus, *C. diphtheriae* [11], was included. Species with at least one publicly available genome sequence (not necessarily the type strain) are highlighted in **bold face**. The tree is based on sequences aligned by the RDP aligner, utilizes the Jukes-Cantor corrected distance model to construct a distance matrix based on alignment model positions without the use of alignment inserts, and uses a minimum comparable position of 200. The tree is built with RDP Tree Builder, which uses Weighbor [12] with an alphabet size of 4 and length size of 1,000. The building of the tree also involves a bootstrapping process repeated 100 times to generate a majority consensus tree [13]. *Rhodococcus equi* (X80614) was used as an outgroup.

Figure 1 shows the phylogenetic neighborhood of *C. halotolerans* in a 16S rRNA based tree. The sequences of the four identical 16S rRNA gene copies in the genome differ by eight nucleotides from the previously published 16S rRNA sequence (AY226509), which contains two ambiguous bases.

**Figure 2:**
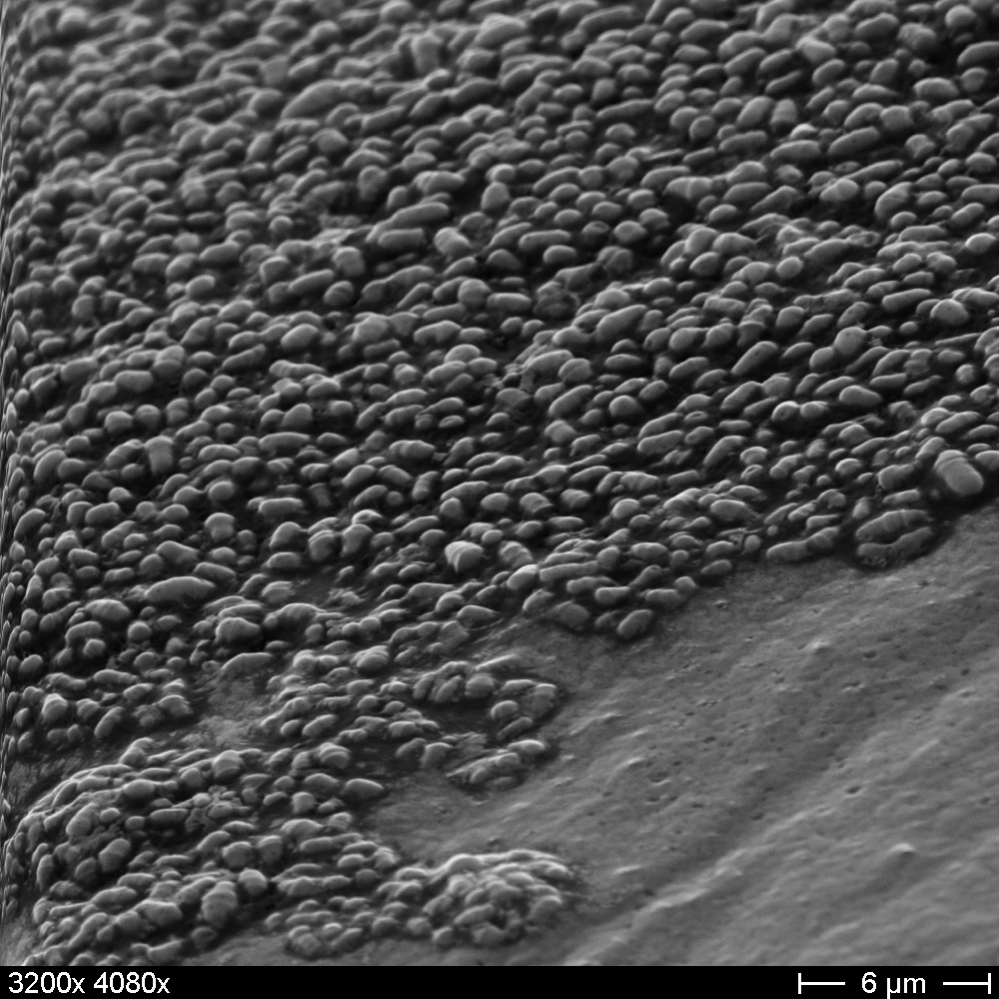
Scanning electron micrograph of *C. halotolerans* YIM 70093^T^.

*C. halotolerans* YIM 70093^T^ is Gram-positive and cells are rod-shaped, 0.5-1 *μ*m long and 0.25-0.5 *μ*m wide (Table 1 and Figure 2). It is described to be non-motile [1], which coincides with a complete lack of genes associated with ‘cell motility’ (functional category N). Optimal growth of YIM 70093^T^ was shown to occur at 28°C, pH 7.2 and 100 g/l KCl, albeit the strain tolerates a wide range of salinity, between 0-250 g/l, NaCl, and MgCl_2_ [1]. Carbon sources utilized by strain YIM 70093^T^ include glucose, galactose, sucrose, arabinose, mannose, mannitol, maltose, xylose, ribose, salicin, dextrin, and starch [1], although the latter is doubtful as *C. halotolerans* cannot hydrolize starch [1].

**Table 1:** Classification and general features of *C. halotolerans* YIM 70093^T^ according to the MIGS recommendations [14].

**MIGS ID**	**Property**	**Term**	**Evidence code^a)^**
	Current classification	Domain *Bacteria*	TAS [15]
		Phylum *Actinobacteria*	TAS [16]
		Class *Actinobacteria*	TAS [17]
		Order *Actinomycetales*	TAS [17-20]
		Family *Corynebacteriaceae*	TAS [17,18,20,21]
		Genus *Corynebacterium*	TAS [18,22,23]
		Species *Corynebacterium halotolerans*	TAS [1]
		Type-strain YIM 70093 (=DSM 44683)	TAS [1]
	Gram stain	Positive	TAS [1]
	Cell shape	diphtheroid, irregular rods	TAS [1]
	Motility	non-motile	TAS [1]
	Sporulation	non-sporulating	TAS [1]
	Temperature range	Mesophile	NAS
	Optimum temperature	28°C	TAS [1]
	Salinity	0-250 g/l KCl/NaCl/MgCl_2_	TAS [1]
MIGS-22	Oxygen requirement	Aerobe	TAS [1]
	Carbon source	glucose, galactose, sucrose, arabinose, mannose, mannitol, maltose, starch, xylose, ribose, salicin, dextrin	TAS [1]
	Energy metabolism	Chemoorganoheterotroph	TAS [1]
	Terminal electron acceptor	Oxygen	NAS
MIGS-6	Habitat	saline soil	TAS [1]
MIGS-15	Biotic relationship	free living	NAS
MIGS-14	Pathogenicity	non-pathogenic	NAS
	Biosafety level	1	TAS [24]
MIGS-23.1	Isolation	saline soil	TAS [1]
MIGS-4	Geographic location	Xinjiang Province, China	TAS [1]
MIGS-5	Sample collection time	Not reported	
MIGS-4.1	Latitude	Not reported	
MIGS-4.2	Longitude	Not reported	
MIGS-4.3	Depth	Not reported	
MIGS-4.4	Altitude	Not reported	

a) Evidence codes - TAS: Traceable Author Statement (i.e., a direct report exists in the literature); NAS: Non-traceable Author Statement (i.e., not directly observed for the living, isolated sample, but based on a generally accepted property for the species, or anecdotal evidence). These evidence codes are from the Gene Ontology project [25].

### Chemotaxonomy

The peptidoglycan of strain YIM 70093^T^ contains *meso*-diaminopimelic acid, galactose, and arabinose [1], therefore it belongs to cell wall type IV, sugar type A. The menaquinones detected in the cell membrane of YIM 70093^T^ are MK-8(H_2_) (35.5%) and MK-9(H_2_) (64.5%) [1]. Cellular fatty acids are predominantly saturated straight chain acids, C_16:0_ (42.1%), C_14:0_ (7.3%); and C_18:0_ (4.5%), and unsaturated acids, *cis*-9-C_18:1_ (28.9%) and *cis*-9-C_16:1_ (9.8%), in addition to 10-methyl C_18:0_ (7.4%) [1]. Like many, but not all corynebacteria, *C. halotolerans* also contains mycolic acids, predominantly of the short chain type (C_32_-C_36_): C_32:0_ (36.0%), C_34:0_ (20.8%), C_34:1_ (25.1%), C_36:0_ (3.6%), C_36:1_ (8.4%), and C_36:2_ (5.1%) [1]. The reported major polar lipids consist of diphosphatidylglycerol (DPG), phosphatidylglycerol (PG), phosphatidylinositol (PI), glycolipid and phosphatidylinositol mannosides (PIM) [1].

## Genome sequencing and annotation

### Genome project history

*C. halotolerans* YIM 70093^T^ was selected for sequencing as part of a project to define the core genome and pan genome of the non-pathogenic corynebacteria due to its phylogenetic position and interesting capabilities, i.e. high salt tolerance. While not being a part of the *** G****enomic* ***E****ncyclopedia of* ***Bacteria**** and* ***Archaea***** (GEBA) project [26], sequencing of the type strain will nonetheless aid the GEBA effort. The genome project is deposited in the Genomes On Line Database [27] and the complete genome sequence is deposited in GenBank. Sequencing, finishing and annotation were performed by the Center of Biotechnology (CeBiTec). A summary of the project information is shown in Table 2.

**Table 2:** Genome sequencing project information

**MIGS ID**	**Property**	**Term**
MIGS-31	Finishing quality	Finished
MIGS-28	Libraries used	Two genomic libraries: one 454 pyrosequencing PE library (3.2 kb insert sizes), one Illumina library
MIGS-29	Sequencing platforms	454 GS FLX Titanium, Illumina GA IIx
MIGS-31.2	Sequencing coverage	22.5 × Pyrosequencing; 23.5 × SBS
MIGS-30	Assemblers	Newbler version 2.3
MIGS-32	Gene calling method	GeneMark, Glimmer
	INSDC ID	CP003697, CP003698
	GenBank Date of Release	July 1, 2013 / after publication
	GOLD ID	Gi19308
	NCBI project ID	168616
MIGS-13	Source material identifier	DSM 44683
	Project relevance	Industrial, GEBA

### Growth conditions and DNA isolation

*C. halotolerans* strain YIM 70093^T^, DSM 44683, was grown aerobically in CASO broth (Carl Roth GmbH, Karlsruhe,Germany) at 30°C. DNA was isolated from ~ 10^8^ cells using the protocol described by Tauch *et al*. 1995 [28].

### Genome sequencing and assembly

The genome was sequenced using a 454 sequencing platform. A standard 3k paired end sequencing library was prepared according to the manufacturers protocol (Roche). Pyrosequencing reads were assembled using the Newbler assembler v2.3 (Roche). The initial Newbler assembly consisted of 81 contigs in six scaffolds with an additional 26 lone contigs. Analysis of the six scaffolds revealed one to be an extrachromosomal element (plasmid pCha1), four to make up the chromosome with the remaining one to contain the four copies of the RRN operon which caused the scaffold breaks. The scaffolds were ordered based on alignments to the complete genomes of *C. glutamicum* [29] and *C. efficiens* [30] and subsequent verification by restriction digestion, Southern blotting and hybridization with a 16S rDNA specific probe.

The Phred/Phrap/Consed software package [31-34] was used for sequence assembly and quality assessment in the subsequent finishing process. After the shotgun stage, gaps between contigs were closed by editing in Consed (for repetitive elements) and by PCR with subsequent Sanger sequencing (IIT Biotech GmbH, Bielefeld, Germany). A total of 61 additional reactions were necessary to close gaps not caused by repetitive elements. To raise the quality of the assembled sequence, Illumina reads were used to correct potential base errors and increase consensus quality. A WGS library was prepared using the Illumina-Compatible Nextera DNA Sample Prep Kit (Epicentre, WI, U.S.A) according to the manufacturer's protocol. The library was sequenced in an 80 bp single read GA*IIx* run, yielding 1,497,321 total reads. Together, the combination of the Illumina and 454 sequencing platforms provided 46.0× coverage of the genome.

### Genome annotation

Gene prediction and annotation were done using the PGAAP pipeline [35]. Genes were identified using GeneMark [36], GLIMMER [37], and Prodigal [38]. For annotation, BLAST searches against the NCBI Protein Clusters Database [39] were performed and the annotation was enriched by searches against the Conserved Domain Database [40] and subsequent assignment of coding sequences to COGs. Non-coding genes and miscellaneous features were predicted using tRNAscan-SE [41], Infernal [42], RNAMMer [43], Rfam [44], TMHMM [45], and SignalP [46].

## Genome properties

**Figure 3:**
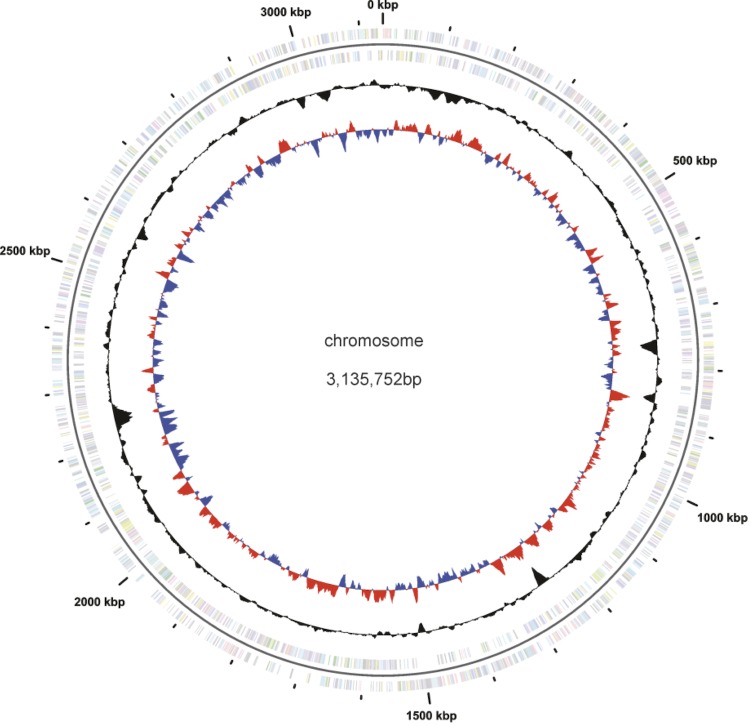
Graphical map of the chromosome (not drawn to scale with plasmid). From the outside in: Genes on forward strand (color by COG categories), Genes on reverse strand (color by COG categories), GC content, GC skew.

**Figure 4:**
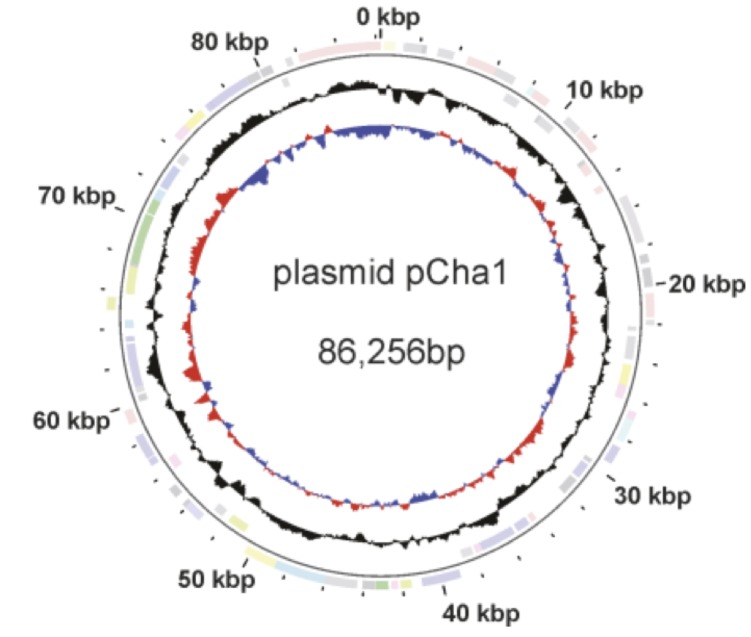
Graphical map of the plasmid pCha1 (not drawn to scale with chromosome). From the outside in: Genes on forward strand (color by COG categories), genes on reverse strand (color by COG categories), GC content, GC skew.

The genome includes one plasmid, for a total size of 3,222,008 bp, with one circular chromosome of 3,135,752 bp (68.44% G+C content) and one plasmid of 86,256 bp (63.20% G+C content) [Figure 3 and Figure 4]. For the main chromosome, 2,856 genes were predicted, 2,791 of which are protein-coding genes. 1,632 (57%) of the protein-coding genes were assigned to a putative function with the remaining annotated as hypothetical proteins. 1,914 protein coding genes belong to 396 paralogous families in this genome corresponding to a gene content redundancy of 66.8%. The properties and the statistics of the genome are summarized in Table 3, Tables 4 and 5.

**Table 3:** Summary of genome: one chromosome and one plasmid

**Label**	**Size (Mb)**	**Topology**	**INSDC identifier**
Chromosome	3.136	circular	CP003697.1
Plasmid pCha1	0.086	circular	CP003698.1

**Table 4:** Genome Statistics

**Attribute**	**Value**	**% of total^a^**
Genome size (bp)	3,222,008	100.00%
DNA coding region (bp)	2,791,134	86.63%
DNA G+C content (bp)	2,200,760	68.30
Total genes^b^	2,930	100.00%
RNA genes	65	2.22%
rRNA operons	4	
tRNA genes	53	1.81%
Protein-coding genes	2,865	97.78%
Genes with function prediction (protein)	1,632	56.96%
Genes assigned to COGs	2,234	77.98%
Gene in paralog clusters	1,914	66.81%
Genes with signal peptides	251	8.76%
Genes with transmembrane helices	686	23.94%

a) The total is based on either the size of the genome in base pairs or the total number of protein coding genes in the annotated genome.

**Table 5:** Number of genes associated with the general COG functional categories

**Code**	**Value**	**%age**	**Description**
J	155	5.41	Translation, ribosomal structure and biogenesis
A	1	0.03	RNA processing and modification
K	185	6.46	Transcription
L	141	4.92	Replication, recombination and repair
B	0	0.00	Chromatin structure and dynamics
D	20	0.70	Cell cycle control, cell division, chromosome partitioning
Y	0	0.00	Nuclear structure
V	44	1.54	Defense mechanisms
T	81	2.83	Signal transduction mechanisms
M	126	4.40	Cell wall/membrane biogenesis
N	0	0.00	Cell motility
Z	0	0.00	Cytoskeleton
W	0	0.00	Extracellular structures
U	25	0.87	Intracellular trafficking and secretion, and vesicular transport
O	88	3.07	Posttranslational modification, protein turnover, chaperones
C	176	6.14	Energy production and conversion
G	183	6.39	Carbohydrate transport and metabolism
E	262	9.14	Amino acid transport and metabolism
F	68	2.37	Nucleotide transport and metabolism
H	122	4.26	Coenzyme transport and metabolism
I	88	3.07	Lipid transport and metabolism
P	196	6.84	Inorganic ion transport and metabolism
Q	85	2.97	Secondary metabolites biosynthesis, transport and catabolism
R	360	12.57	General function prediction only
S	214	7.47	Function unknown
-	631	22.02	Not in COGs
